# The Plk1 Inhibitor BI 2536 Temporarily Arrests Primary Cardiac Fibroblasts in Mitosis and Generates Aneuploidy *In Vitro*

**DOI:** 10.1371/journal.pone.0012963

**Published:** 2010-09-24

**Authors:** Bo Lu, Hasan Mahmud, Alexander H. Maass, Bo Yu, Wiek H. van Gilst, Rudolf A. de Boer, Herman H. W. Silljé

**Affiliations:** 1 Department of Cardiology, University Medical Center Groningen, University of Groningen, Groningen, The Netherlands; 2 Department of Cardiology, The Second Affiliated Hospital of Harbin Medical University, Key Laboratories of Education Ministry for Myocardial Ischemia Mechanism and Treatment, Harbin, China; Roswell Park Cancer Institute, United States of America

## Abstract

BI 2536 is a new anti-mitotic drug that targets polo-like kinase 1 (Plk1) and is currently under clinical development for cancer therapy. The effect of this drug on cancer cells has been extensively investigated, but information about the effects on primary dividing cells and differentiated non-dividing cells is scarce. We have investigated the effects of this drug on primary neonatal rat cardiac fibroblasts and on differentiated cardiomyocytes and explored the possibility to use this drug to enrich differentiated cell populations *in vitro*. BI 2536 had a profound effect on cardiac fibroblast proliferation *in vitro* and arrested these cells in mitosis with an IC50 of about 43 nM. Similar results were observed with primary human cells (HUVEC, IC50  = 30 nM), whereas the cancer cell line HeLa was more sensitive (IC50 of 9 nM). Further analysis revealed that prolonged mitotic arrest resulted in cell death for about 40% of cardiac fibroblasts. The remaining cells showed an interphase morphology with mostly multi- and micro-nucleated nuclei. This indicates that a significant number of primary fibroblasts are able to escape BI 2536 induced mitotic arrest and apparently become aneuploid. No effects were observed on cardiomyocytes and hypertrophic response (growth) upon endothelin-1 and phenylephrine stimulation was normal in the presence of BI 2536. This indicates that BI 2536 has no adverse effects on terminally differentiated cells and still allows proliferation independent growth induction in these cells. In conclusion, cardiomyocytes could be enriched using BI 2536, but the formation of aneuploidy in proliferating cells most likely limits this *in vitro* application and does not allow its use in putative cell based therapies.

## Introduction

Contamination with proliferating cells is often a serious problem in cell culture studies investigating differentiated or quiescent cell populations, since the former can easily overgrow the cell type of interest. Examples include cardiomyocyte and neurological cell research, while also in the field of stem cell differentiation for cell therapies this represents a common problem. To remove proliferating cells from differentiated cell populations, nucleotide analogues are often used like bromodeoxyuridine (BrdU) and arabinoside [1], [2], which are incorporated in the DNA of proliferating cells resulting in DNA damage checkpoint activation and cell cycle arrest. Since these drugs affect the genetic code they cannot be used in any subsequent therapy. Moreover, these analogues are also incorporated in mitochondrial DNA and can interfere with mitochondrial biogenesis of the differentiated cell population. Other methods, like FACS analysis often require specific antibodies and the throughput is often limited. With the development of more specific anti-cancer drugs, we decided to investigate the potential of the Polo-like kinase 1 (PLK1) inhibitor BI 2536 [3], [4] as a potential drug to eliminate proliferating cells from *in vitro* cultures containing terminally differentiated cardiomyocytes.

Polo-like kinase 1 (Plk1) is a mitotic kinase, which is highly expressed in proliferating cells only during the G2 and M phase of the cell cycle. It has specific roles during mitotic progression, including centrosome maturation, spindle assembly, chromosome segregation and cytokinesis [5], [6]. Plk1 consist of two domains, a C-terminal catalytic kinase domain and a N-terminal polo-box-domain (PBD), which recognizes specific phosphorylated targeting sequences [7], [8] and is essential for its specific localisation and interaction with some of its substrates [7], [9], [10]. Micro-injection of Plk1 antibodies and siRNA based studies targeting Plk1 have shown the essential role of Plk1 in mitotic progression in cancer cells [11]–[15]. These studies revealed that functional interference with Plk1 resulted in a mitotic arrest with condensed chromosomes, monopolar spindles and non-matured centrosomes. Recent studies with small molecules targeting Plk1, have confirmed these effects, and moreover revealed late stage mitotic functions for Plk1 [3], [16]. Following prolonged mitotic arrest, cell death (apoptosis) is induced in Plk1-inhibited cancer cells and hence Plk1 has been proposed to be a promising anti-cancer target [4], [17].

Despite the large body of evidence in cancer cells, the role of Plk1 in primary cells has only been poorly investigated and conflicting results have been published. In primary fibroblasts, Plk1 antibody microinjection was shown to arrest these cells with a G2-like phenotype, in contrast to the mitotic arrest in cancer cells [11]. G2-phase functions for Plk1, including recovery from DNA damage checkpoints, are supported by several studies [18]–[21]. Nevertheless, the role of Plk1 in G2-M phase transition in mammalian cells under normal conditions is still under debate [5]. Also siRNA studies showed different results in normal cells, as compared to cancer cells. In direct comparisons normal cells did not appear to be affected by Plk1 depletion, whereas cancer cells arrested in mitosis followed by cell death [12], [13]. Together with the facts that numerous cancer cells show increased Plk1 expression, these results further supported that Plk1 could be a specific anti-cancer target [17].

BI 2536 is currently the most intensively investigated Plk1 inhibitor [22]. BI 2536 is a small molecule inhibiting Plk1 at sub-nanomolar concentrations in vitro (0.83 nM) and is equipotently active against human, mouse and rat Plk1 [4]. It was shown over 1000-fold more selective towards Plk1 as compared to 63 other kinases and only some activity against the closely related kinases Plk2 and Plk3 were reported [3]. BI2536 has been tested on numerous human cancer cell lines *in vitro*, and tumor Xenograft models suggested prominent anti-cancer activity [3]. Several phase I and phase II trials have been performed [22]–[24], but its potential as an anti-cancer target still has to be established. The latest phase II trial study on five different cancer types was rather disappointing, showing only limited anti-tumor activity [24].

Here we tested the potential *in vitro* application of this drug by studying the elimination of proliferating cells from cultures containing primary differentiated cells. We used a commonly used system in cardiac research consisting of partially purified primary differentiated neonatal cardiomyocytes. We describe the effects of BI 2536 on these differentiated cells as well as on primary cardiac fibroblasts and show detrimental effects on primary proliferating cells.

## Methods

### Reagents and chemicals

Cell culture medium and serum were obtained from Lonza Benelux B.V. (Breda, The Netherlands) and penicillin-streptomycin and trypsin were from Invitrogen (The Netherlands). Mouse monoclonal anti-α-actinin antibody EA53 and mouse monoclonal anti-α-tubulin (B-5-1-2) antibody were from Sigma-Aldrich Chemie B.V., rabbit polyclonal anti-Troponin (ab9332) was from Abcam (Cambridge, MA, USA), FITC labelled anti-Phospho-Histone H3 (Ser10) antibody was from Cell Signaling. Antibody Dylight 649 labelling kit was from Pierce Biotechnology (Rockford, IL, USA) BI2536 was purchased from Axon Medchem (Groningen, The Netherlands) and dissolved in DMSO at a concentration of 100 µM. L-[4,5-^3^H]Leucine (37 MBq/ml; 5,85 TBq/mmol) was from GE Healthcare Europe (Diemen, Belgium) and Ultima Gold XR scintillation liquid was from Perkin Elmer (Groningen, The Netherlands). Unless otherwise stated all chemicals were purchased at the highest possible grade from Sigma-Aldrich Chemie B.V. (Zwijndrecht, The Netherlands).

### Neonatal rat cardiac myocyte and fibroblast isolation

Neonatal rat ventricle myocytes were isolated from the cardiac ventricles of 1–3 days old Sprague-Dawley pups. For isolation we followed a previously described protocol [1], [25]. In short, hearts were removed from the thoracic cavity and placed in a tube containing cold CBFHH solution (137 mM NaCl, 5.36 mM KCL, 0.81 mM MgSO4, 5.55 mM dextrose,0.44 mM KH2PO4, 0.34 mM Na2HPO4, 20 mM HEPES, pH 7.4.). Ventricles were separated from other tissue using scissors and cut into several pieces. Subsequently cardiomyocytes and fibroblasts were detached from the extracellular matrix by repeated incubation in CBFHH, supplemented with 2 mg/ml trypsin and 20 µg/ml DNase. Cells were collected by centrifugation and tissue clumps were removed by filtration. Subsequently, cells were preplated in cell culture dishes in 50 ml MEM medium with 5% FCS for 45 min. During this period most non-cardiomyocyte cells (mainly fibroblasts) attached to the dish, whereas cardiomyocytes remained in solution. The cardiomyocytes were subsequently transferred to separate tissue culture dishes and allowed to attach. Both fibroblasts and cardiomyocytes were subsequently cultured in DMEM medium containing 10% FCS.

All animal studies were conducted in accordance with the NIH Guide for the Care and Use of Laboratory Animals and approved by the Committee for Animal Experiments of the University of Groningen (Approval ID: DEC 5495).

### Cell culture

Neonatal rat primary cardiac fibroblasts and cardiomyocytes and HeLa (S3) were cultured at 37°C under 5% CO_2_ in DMEM medium, supplemented with 10% FCS and penicillin-streptomycin (100 IU/ml and 100 µg/ml, respectively). BI was added to a final concentration of 100 nM, unless otherwise stated. Corresponding control cultures received an equal volume of solvent (DMSO). Primary human umbilical vein endothelial cells (HUVEC) were obtained from the Endothelial Cell Facility (University Medical Center Groningen, The Netherlands). HUVEC were isolated from two umbilical cords and prepared as previously described [26], [27]. Cells were cultured on 1% pre-coated gelatin plates at 37°C under 5% CO_2_ in RPMI 1640 medium was supplemented with 20% FCS, 2 mmol/l L-Glutamine, 5 U/ml heparin, 100 IU/ml penicillin, 100 µg/ml streptomycin and 50 µg/ml endothelial cell growth factor extracted from bovine brain. HUVEC experiments were conducted with cell passage numbers between 2 and 6.

### FACS analysis

For FACS analysis attached cells were detached by trypsin treatment and collected by centrifugation together with free floating cells in the medium. After a PBS wash cells were fixed in 4% paraformaldehyde for 10 min at 4°C. Cells were subsequently washed with PBS and stored at 4°C in PBS until all samples from one experiment were collected. Subsequently cells were treated with ice cold PBS + 0.1% tritonX100 for 5 min and washed again with PBS. Cells were subsequently incubated in 100 µl PBS+1% BSA containing the indicated antibodies and propridium iodide (1 µg/ml) for 30 min at room temperature. FITC labeled anti-Phospho-Histone H3 (Ser10) antibody was used at a 1∶200 dilution. Anti α-actinin antibody was first labelled with DyLight 649, according to the manufacturer instructions (Pierce Biotechnology, Rockford, IL, USA). This labelled antibody was then used at a 1∶100 dilution. FACS analysis was performed on a FACS calibur (Becton Dickinson) and data were analysed with WinMDI2.9.

### Quantitative Real-Time PCR

Relative expression of ANP, Troponin and Periostin genes was determined by quantitative PCR (qPCR). Gene expression was determined by correcting samples for reference gene values (cyclophilin A), and values were expressed relative to the control group per experiment. Primer sequences were: Troponin T-1, cagaagaggttggtcctgatgaa; Troponin T-2, gcaccaagttgggcatgaa; Periostin-1, gatccacggagaaccagtc; Periostin-2, cccacctcctgtggaaatc; Cyclophilin-1, cctcataccagcgacgattc; Cyclophilin-2, atgtggaggagtctcacttc.

### Microscopy

For cardiomyocytes, coverslips were coated with 1 µg/cm^2^ laminin (Millipore) for at least three hours. For fibroblasts non-coated coverslips were used and HUVEC cells were grown on coverslips coated with poly-lysine and 1% gelatin. Cells were grown on coverslips for the indicated time period and subsequently washed with PBS and fixed in paraformaldehyde solution for 10 min at RT. Cells were then permeabilised with ice cold PBS+0.5% Triton X100 for 5 min and then washed with PBS. Antibody incubations were performed in PBS+1%BSA for 1 hour at room temperature. Primary antibodies used were: mouse monoclonal anti-α-tubulin and rabbit anti-troponin. Secondary antibodies were: goat anti-mouse Alexa555 (2 µg/ml) and goat anti-rabbit Alexa488 (2 µg/ml) and nuclei were stained with TO-PRO3 (Invitrogen-Molecular Probes). Normal overview pictures and movies were made on a Leica DMIL microscope equipped with a Leica camera. Confocal images were made on a Leica SP2 AOBS confocal microscope. All images were processed with ImageJ 1.43 M (NIH, USA) and Adobe Photoshop 7.01.

### [^3^H]-Leucine incorporation assay

Cells were grown in 12 well plates and subsequently starved for 24 h in DMEM containing starvation medium lacking FCS. As described previously [28], L-[4,5-^3^H]Leucine was added to all wells and cells were cultured for an additional 20 hours either in the presence or absence of 10 nM endothelin-1 (ET-1) or 50 µM phenylephrine (PE). Subsequently, cells were washed twice with 1 ml PBS, followed by an incubation for 1 hour at 4°C in 1 ml cold 5% TCA was. After one wash with TCA solution and one wash with PBS, proteins were solubilised with 0.5 ml 0.5 M NaOH for 1 h at RT and transferred to scintillation tubes containing 5 ml Ultima Gold XR scintillation liquid. The amount of radioactivity (CPM) was determined by a LS6500 Beckman Coulter scintillation counter.

## Results

### BI 2536 treatment of primary cardiomyocyte cultures

**Figure 1:**
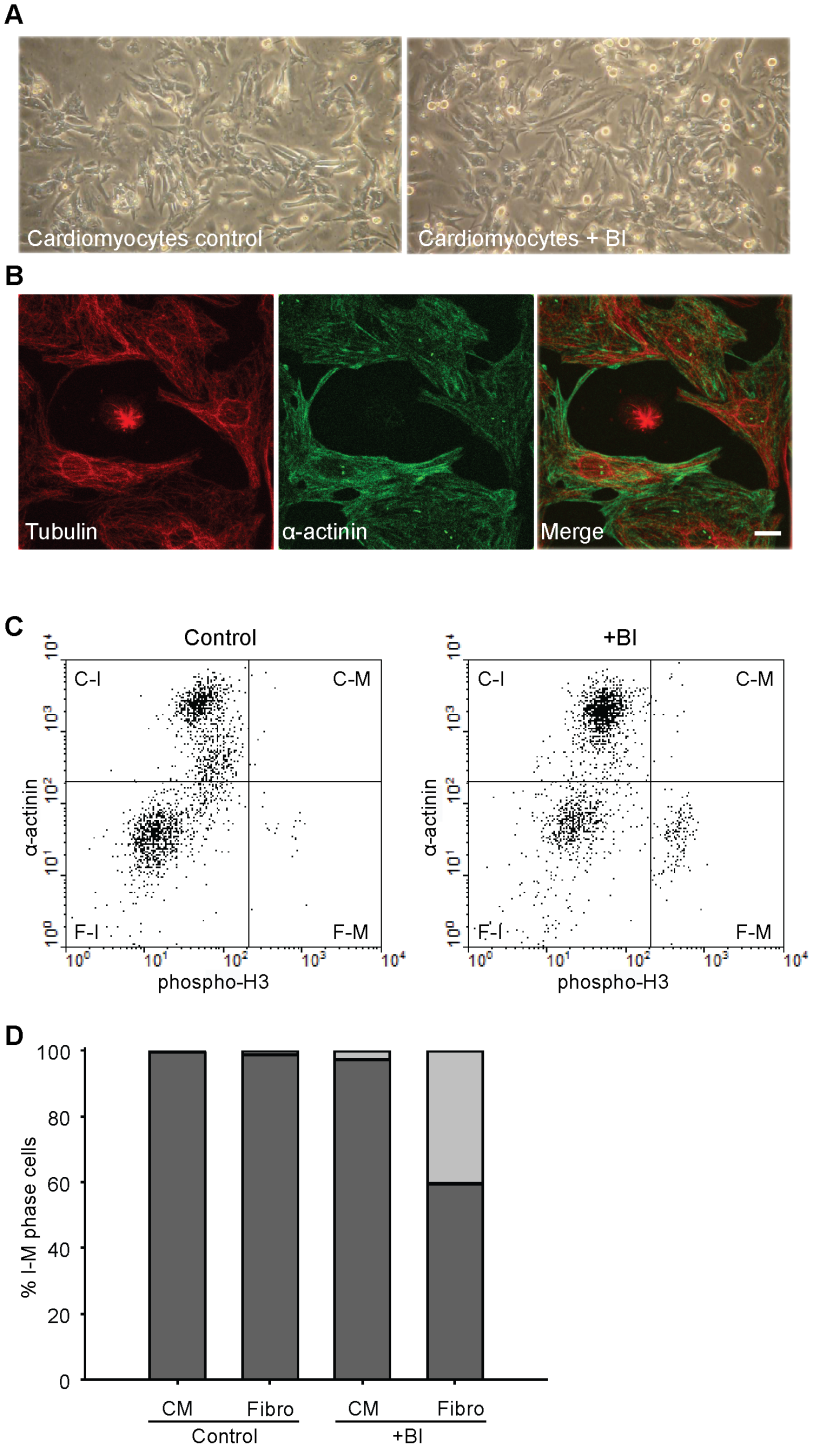
Effect of BI 2536 on neonatal rat cardiomyocytes. Neonatal rat cardiomyocytes were cultured for three days before 100 nM BI 2536, or DMSO as control, was added. A. Light microscopy pictures of control and 24 hours BI 2536 treated cultures. B. Immunofluorescence microscopy pictures of BI 2536 treated cells stained with anti-troponinT (red) and anti-α-tubulin (green). In the middle a mitotic cell is shown with monopolar spindle devoid of troponin T staining. Scale bar is 10 µM. C. FACS analysis of 24 h treated and untreated cells stained with anti-α-actinin and anti-Phospho-Histone H3. Four regions are selected and marked as follow: C-I =  cardiomyocytes in interphase, C-M  =  cardiomyocytes in mitosis, F-I =  fibroblasts in interphase, F-M  =  fibroblasts in mitosis. D. Quantification of FACS profiles of control and 24 h BI 2536 (BI) treated cells stained like in C. Dark grey bars indicate the percentage interphase cells and light grey bars indicate the percentage mitotic cells. CM =  cardiomyocyte, Fibro =  fibroblast.

Primary cardiomyocytes were isolated from neonatal rat hearts and were cultured for three days in the presence of serum in order to adapt to the medium and allow any cardiomyocytes that have not reached end-stage differentiation to fulfil a last round of division. Subsequently, cardiomyocyte cultures were treated with the Plk1 inhibitor BI 2536 or were left untreated. As shown in figure 1A, addition of 100 nM BI 2536, a concentration shown to arrest most cell lines maximally [4], resulted in a small increase in rounded up cells within 24 hours, indicative for a mitotic arrest. Further analysis by immunofluorescence microscopy confirmed that these rounded cells were in mitosis and contained predominantly monopolar spindles with condensed mitotic chromosomes (Fig. 1B and data not shown). This is similar to what has been shown for cancer cell lines. Co-staining with troponin-T, a cardiomyocyte specific marker revealed that the arrested cells were not cardiomyocytes and cardiomyocytes all showed normal interphase morphology (Fig. 1B). To further confirm this, FACS analysis was performed using phospho-Histone H3 as a mitotic marker and α -actinin as a cardiomyocyte marker. In the control culture almost all cells were phospho-Histone H3 negative, and hence in interphase figure 1C–D. Moreover, a significant proportion of cells were alpha-actinin negative, indicating the presence of non-cardiomyocytes. This relative high level of these cells is most likely a result of the three days cultivation without inhibitors. Importantly, after 24 hours BI 2536 treatment a significant portion of cells became phospho-Histone H3 positive and these were almost all α -actinin negative (Fig. 1C–D). This shows that cardiomyocytes were not sensitive to BI 2536 and that predominantly the non-cardiomyocyte cells were targeted by BI 2536.

### BI 2536 does not affect cardiomyocyte function and cellular growth (hypertrophy)

Next we addressed if cardiomyocyte function could be affected by BI 2536. We first examined the beating frequency of these cells by video imaging. As shown in the supplemental videos beating frequency in BI 2536 treated cultures (File S1) was similar to control cultures (File S2). Also beta-adrenergic stimulation by isoproteronol was still normal in BI 2536 treated cells (data not shown). Even after two weeks incubation cardiomyocyte beating was still evident, indicating that BI 2536 does not show apparent interference with this major cardiomyocyte function.

**Figure 2:**
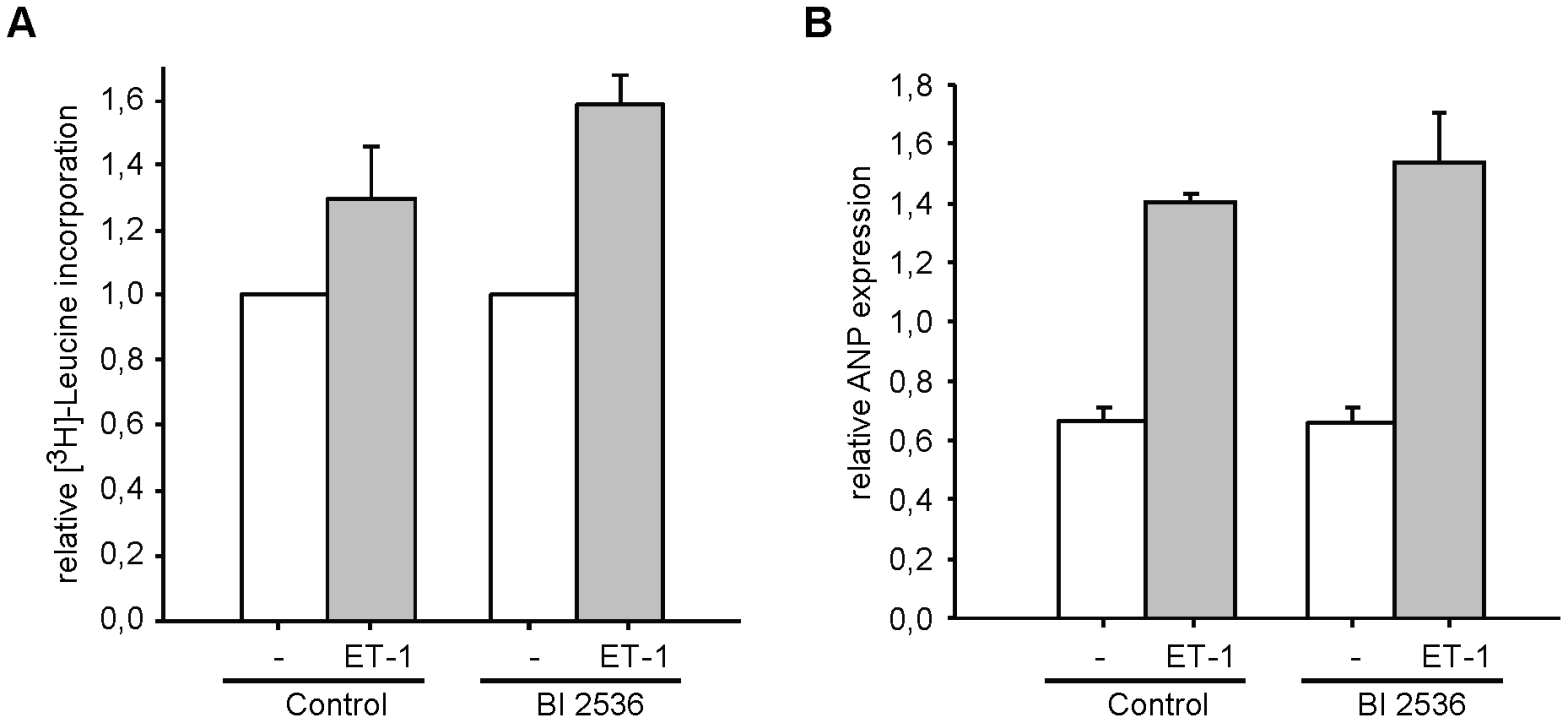
Effect of BI 2536 on hypertrophy induction in neonatal rat cardiomyocytes. Cardiomyocyte cultures were grown as before with or without 100 nM BI 2536 and subsequently serum starved in the presence or absence of BI 2536. A. Effect of treatment with the hypertrophy inducing hormone endothelin-1 on cellular growth ([^3^H]-Leucine incorporation) in the presence or absence of BI. [^3^H]-Leucine incorporation into cellular proteins was determined by liquid scintillation counting. Values are relative to the respective non-treated cultures and error bars depict standard deviations (n = 3). B. Analysis of ANP gene expression by real-time PCR in cultures treated with or without ET-1 in the presence or absence of BI 2536. Levels are corrected for expression of the cyclophilin A housekeeping gene. Error bars depict standard deviations (n = 3).

**Figure 3:**
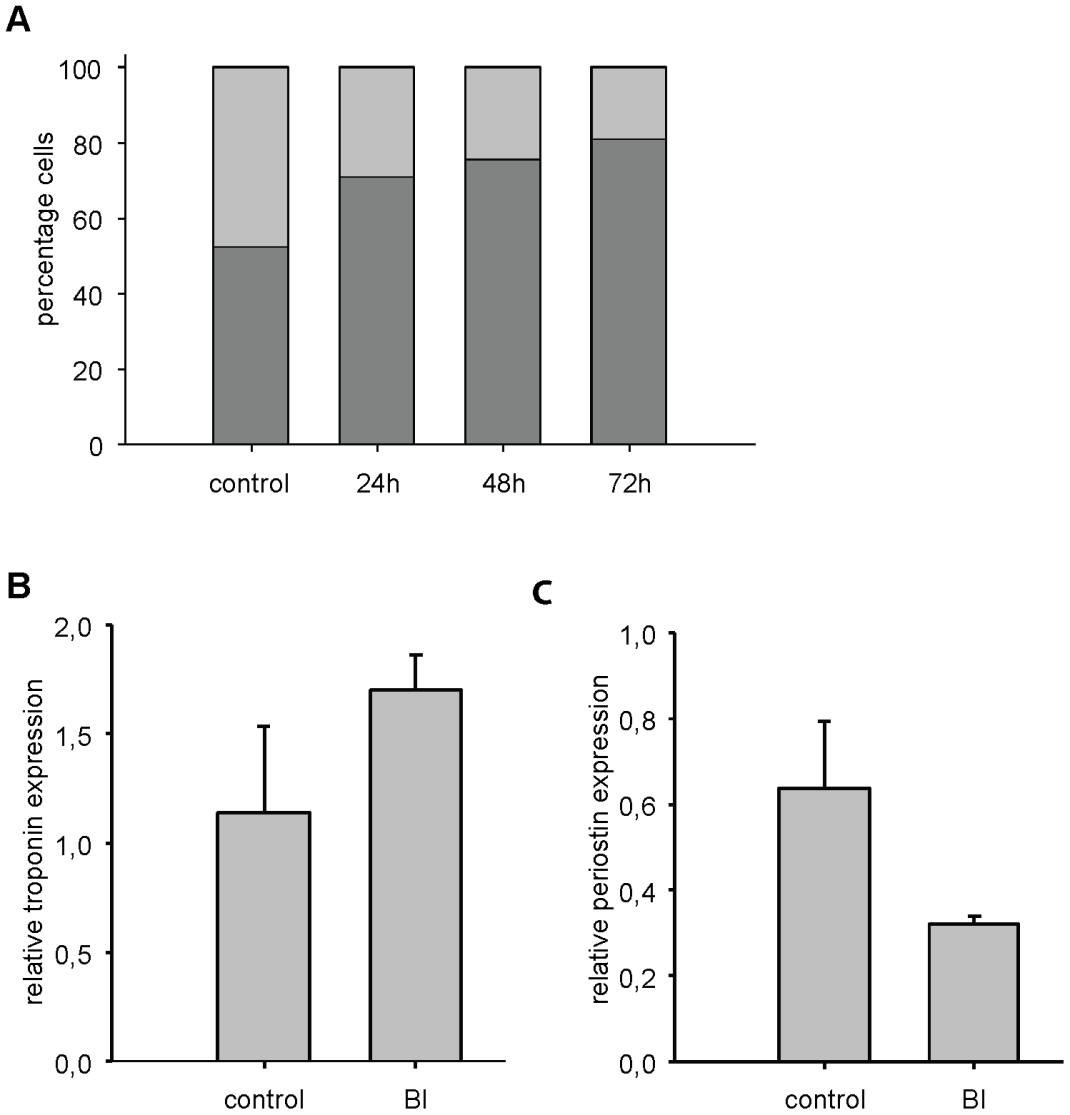
BI 2536 reduces fibroblast numbers in cardiomyocyte cultures. Cardiomyocyte cultures were grown as before with or without 100 nM BI 2536. A) The percentage cardiomyocytes and cardiac fibroblasts were determined by FACS analysis, using anti- α-actinin staining. Before harvest mitotic cells were removed by shake off. Light gray bars indicate fibroblasts, dark grey bars show the percentage cardiomyocytes. B After 24 hour treatment RNA was isolated from cells and real time PCR was performed with primers against cardiac troponin T to determine the expression level of this cardiomyocyte specific gene, providing an indirect measure for the number of cardiomyocytes. Error bars depict standard deviations (n = 3). C. Same as in B, but with primers against periostin, a marker for cardiac fibroblasts.

Primary neonatal cardiomyocyte cultures are predominantly used as a model for cardiac hypertrophy. After two days culture of cardiomyocytes cells were treated with or without BI 2536 for 24 h followed with a similar incubation in starvation medium. Thereafter cells were treated with the hypertrophy inducing stimuli endothelin-1 (ET-1) or phenylephrine (PE) in the presence of [^3^H]Leucine. After 24 hours cells were harvested and the amount of [^3^H]Leucine incorporation into protein was determined. As shown in figure 2A ET-1 strongly increased [^3^H]Leucine incorporation in cardiomyocytes and similar results were observed with PE (data not shown). This effect was even stronger in the presence of BI 2536, indicating that BI 2536 did not interfere with growth induction. The apparent stronger effect in the BI 2536 cultures, is a result of decreased fibroblast numbers in these cultures (see also below and figure 3). To further corroborate this we also investigated ANP gene expression, which is a marker for the hypertrophic response and is expressed in cardiomyocytes only. As shown in figure 2B, ANP expression was similarly up regulated in ET-1 treated cell cultures with and without BI 2536. Together, these results show that BI 2536 does not affect proliferation independent growth inducing responses.

### Not all non-cardiomyocytes can be removed by BI 2536 treatment

The above results showed no apparent interference of cardiomyocytes function in the presence of BI 2536. Next we wanted to know if proliferating cells (>95% fibroblasts) in these cultures could be reduced by BI 2536 treatment. We included a mitotic shake-off to remove most mitotic cells before FACS analysis. As shown in figure 3A, 24 hours BI treatment could already significantly reduce fibroblasts, resulting in a concomitant relative increase in the percentage of cardiomyocytes. This relative increase was further confirmed by the increase of troponin T expression, a cardiomyocytes specific marker, in these cultures (Figure 3B). Conversely expression of the fibroblast marker periostin decreased in these cultures. This further confirmed that only non-cardiomyocytes, predominantly fibroblasts, were affected in these cultures. As shown in figure 3A, prolonged incubation with BI could further lower the percentage of fibroblasts in these cultures. Nevertheless, even after 72 hours, still about 20% non-cardiomyocytes were present as determined by FACS analysis. This is surprising since these cells can perform 2–3 rounds of cell division within this time period (cycling time 28 h). This suggests that not all proliferating cells are properly targeted by BI 2536.

### BI 2536 induces mitotic arrest of primary cardiac fibroblasts

**Figure 4:**
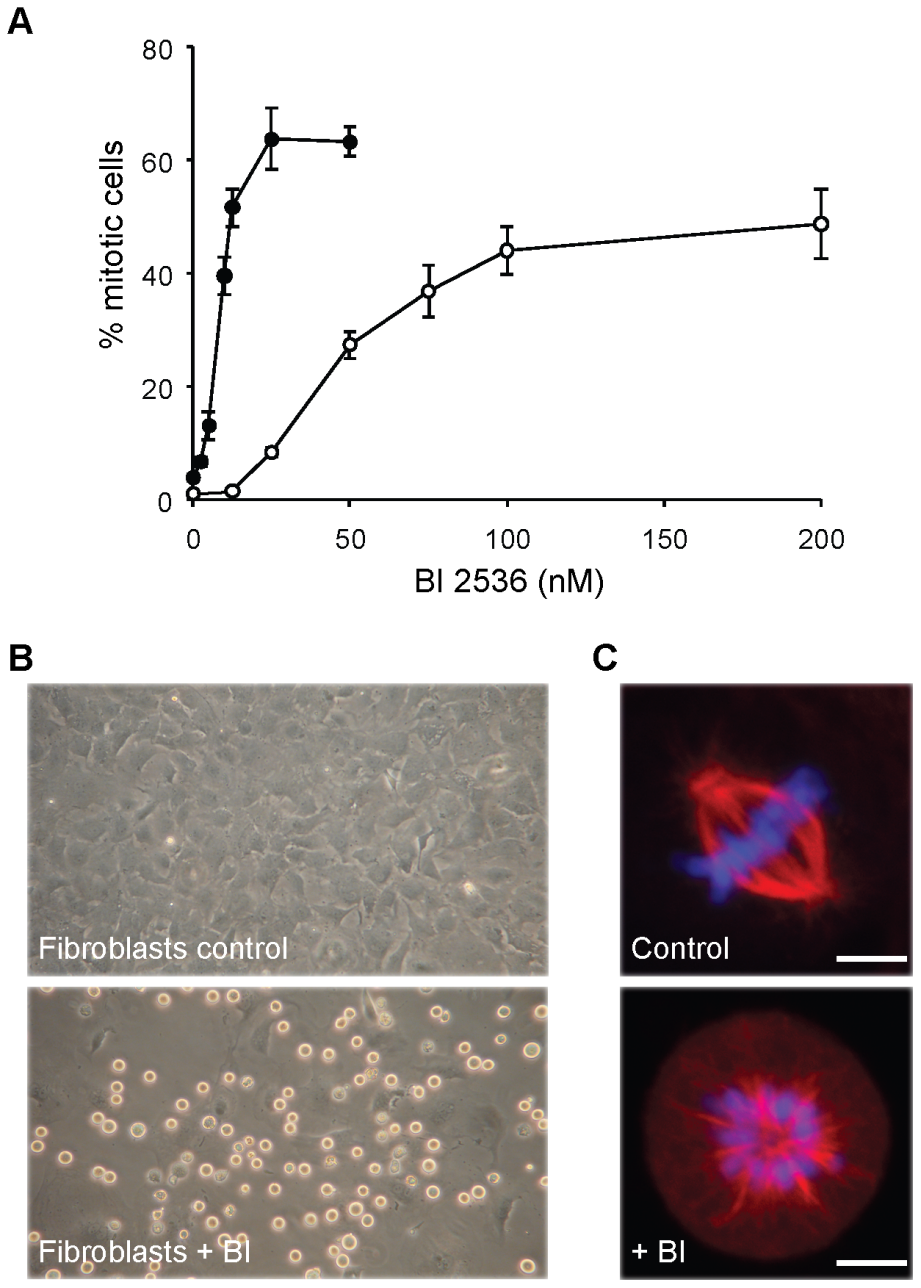
Effect of BI 2536 on rat cardiac fibroblasts. Rat cardiac fibroblasts were cultured for two days before the indicated doses of BI 2536 or DMSO as a control were added. HeLa cells were cultured under identical conditions. A. Dose response curve of primary cardiac fibroblasts and HeLa cells. Cells were cultured for 24 hours with BI 2536 and subsequently fixed and stained with anti-Phospho-Histone H3, as a mitotic marker, and then quantified by FACS analysis. Closed circles, HeLa cells; open circles, cardiac fibroblasts. B. Light microcopy pictures of control and 24 h BI 2536 treated cardiac fibroblast cultures C. Immunofluorescence microscopy pictures of same cells as in B, but stained with anti-α-tubulin (green) and the DNA stain TOPRO-3 (blue). Scale bar is 10 µM.

The contaminating cells in our cardiomyocyte cultures consisted predominantly of cardiac fibroblasts (>95%, data not shown). These cells are easily enriched by preplating and were subsequently used to test the effect of BI 2536 specifically on these primary proliferating cells. Since not all cells appeared to arrest with BI 2536 (figure 3A), we analysed the concentration dependency. Using FACS analysis we determined the number of cells that arrested within 24 hours treatment with different BI 2536 concentrations. This dose response curve revealed that primary fibroblast arrest was maximal at a dose of 100 nM and the IC50 value was approximately 43 nM (Fig. 4A). Not all cells did, however, arrest and a maximum of approximately 50% was observed at concentration of 100 nM and higher. The arrest of cardiac fibroblasts after 24 hours treatment with 100 nM BI 2536 was also clearly evident by the strong increase of rounded up cells (Fig. 4B). Immunofluorescence microscopy confirmed the typical monopolar arrest of these cells, in contrast to the mostly bipolar mitotic cells in the control culture (Fig. 4B). With HeLa cells an IC50 of only 9 nM was observed (Fig. 4A), confirming previous results with these cells, and the maximal percentage of arrested cells was around 63%. At these low concentrations no effects were observed on neonatal rat cardiac fibroblast, showing that these cells are less sensitive. We also tested human primary HUVEC cells which arrested with an (IC50 of 30 nM), indicating that different cell types show different sensitivities.

### BI 2536 temporarily arrests primary cardiac fibroblasts

**Figure 5:**
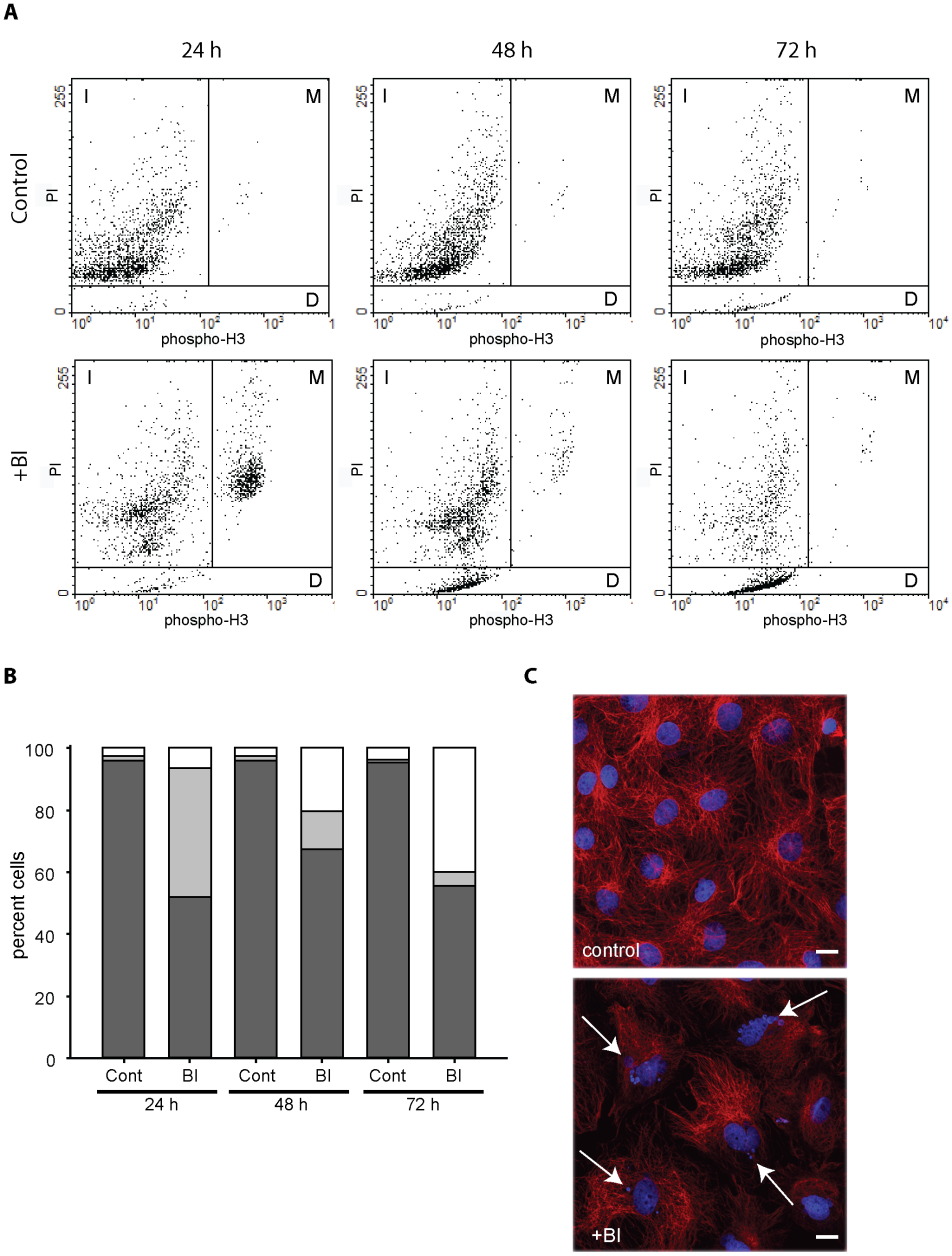
BI 2536 temporarily arrests cardiac fibroblasts. Rat cardiac fibroblasts were cultured for two days before 100 nM BI 2536 or DMSO as control were added. Cells were subsequently analysed at different time points. A. FACS analysis of cells stained with anti-Phospho-Histone H3 and propidium iodide. In the upper panel the control cells are shown and in the lower panel the BI2536 treated cells. The different regions are marked as follow: I is interphase, M is mitosis and D denotes dead cells. B. Quantification of FACS profiles, as shown in A (n = 3). Dark grey bar depict interphase cells, light grey bar depicts mitotic cells and white bar depicts dead cells. C. Immunofluorescence pictures of remaining interphase cells after 72 hours culture in the presence or absence of BI 2536. Cells were stained with anti-α-tubulin (green) and the DNA stain TOPRO-3 (blue). Control cells are shown in the upper panel. Scale bar is 10 µM.

To further corroborate the effect on primary fibroblasts, we also analysed the effect of BI 2536 in time. As shown in figure 5A, 24 hours after treatment with BI2536 a clear mitotic population (phospho-Histone H3 positive) is present, which is absent in the control culture. In time, however, this population is diminishing, and a population of dead, low propidium iodide stained cells became apparent. Prolonged culturing of control cells did only provide a small increase in dead cells. Quantification of FACS experiments (Fig. 5B) revealed that after 24 hours about 41,5% of the cells were in mitosis and that 6,5% cells were dead. After 72 hours this has almost inverted with an average of 4,3% mitotic cells and 40,1% dead cells. Importantly, at this stage still about 55,1% of interphase cells were present. These cells were investigated by immunofluorescence microscopy and it turned out that more than 90% of these cells had multi- or micro-nucleated nuclei (Fig. 5C), a clear sign of aberrant mitotic progression. We did not observe this micro-nucleation in cardiomyocytes treated with BI 2536 (data not shown), which further supports that this only occurred in proliferating cells, most likely due to an escape of the BI 2536 induced mitotic arrest. This also clarifies why BI 2536 treatment does not fully reduce the number of proliferating cells in cardiomyocyte cultures.

## Discussion

In this paper we describe the effect of BI 2536 on primary cardiac fibroblasts and cardiomyocytes. We showed herein that BI 2536 does not generate adverse effects in cardiomyocytes, but does profoundly affect primary fibroblast. In particular, fibroblasts were arrested in mitosis with monopolar spindles and died upon prolonged arrest. Nevertheless part of the cells escaped mitotic arrest, as was evident from the large number of multi- and micro-nucleated cells. Since aneuploidy is a hallmark of most solid cancers, this could be a potential concern for its use and will limit the potential of Plk1 inhibitors for enrichment of differentiated cells for cell therapy. As far as we know, this is the first analysis of the effects of BI 2536 on both primary and differentiated cells.

BI 2536 did not appear to affect the function of differentiated cells, as tested here using neonatal cardiomyocytes. No apparent morphological changes were observed and cardiomyocyte beating continued in the presence of BI 2536. Even after 4 weeks incubation with BI 2536 these cells continued normal beating (data not shown). Despite the fact that these cells cannot proliferate anymore, cardiomyocytes can still increase in size, and cardiomyocyte hypertrophy is one of the hallmarks of heart failure [29], [30]. We therefore also evaluated the effect of BI 2536 on the hypertrophic response to phenylephrine and endothelin-1 *in vitro*. As shown here cardiomyocyte hypertrophy could be induced in the presence of BI 2536, and even appeared more pronounced than in the absence of BI 2536. The latter was most likely due to the reduction in fibroblasts in these BI 2536 treated cultures, resulting in lower background values. Thus, BI 2536 specifically blocks proliferation, but does not seem to affect proliferation-independent hypertrophic cell growth of cardiomyocytes.

Our results with BI 2536 showed a clear mitotic arrest of primary rat fibroblast with monopolar spindles, which is similar to the observed arrest of cancer cells. This is in contrast to anti-Plk1 microinjection study in human fibroblasts, which showed predominantly a G2 phase-like arrest phenotype in primary cells, despite a clear mitotic arrest in cancer (HeLa) cells [11]. Although we cannot exclude a possible G2 delay in these cells, the major phenotype is mitotic arrest. A similar observation was made with primary human (HUVEC) cells, indicating that this is not due to species differences (data not shown). PLK1 siRNA based studies have suggested that Plk1 depletion did not affect normal cells or was much less effective in normal cells, as compared to cancer cells [12], [13]. Although higher concentrations of BI2536 were required to arrest primary rat fibroblasts as compared to HeLa cels, we observed a clear mitotic arrest indicating that also in normal cells Plk1 is essential for proper mitotic progression. The absence of a mitotic phenotype with siRNA studies suggests, therefore, that depletion was not sufficient to generate this phenotype in normal cells.

Like in cancer cells arrested by Plk1 depletion or inhibition, we also observed cell death in primary cells following the mitotic arrest. In addition, however, we observed multi- and micro-nucleated cells in primary fibroblast populations treated with BI2536. After 72 hours even more than 90% of the present interphase fibroblasts showed this multi- and micro-nucleation phenotype. This phenotype is a clear sign of abnormal mitotic progression, indicating that a substantial number of primary fibroblasts escaped the mitotic arrest. This effect has not been reported for cancer cells treated with BI 2536 [4]. This is surprising since it is often thought that, in contrast to normal cells, cancer cells might have a partially impaired mitotic checkpoint, explaining chromosome instability in the latter [31]. Thus, it appears that cancer cells die more efficiently upon Plk1 inhibition, as compared to normal cells. The latter is in line with a recent study describing the effects of microtubule drugs on cancer and normal cells [32]. In this paper, the authors described that cancer cells are more susceptible to cell death upon mitotic arrest by the microtubule stabilizing agent taxol, as compared to normal cells. Our results suggest that this might be similar for Plk1 mediated mitotic arrests, suggesting that this is a general spindle checkpoint associated phenomenon. Although this is beneficial for the elimination of cancer cells, it begs the question what will happen with normal cells that have become aneuploid as a result of BI 2536 treatment. Since aneuploidy is a hallmark of most solid tumors [33] this could be a concern.

Altogether, our results show that Plk1 inhibition by BI 2536 treatment has no obvious effects on differentiated cardiomyocytes. It also, shows that whereas BI 2536 can inhibit proliferation it does not affect proliferation independent cell growth. Plk1 inhibition by BI 2536 resulted in mitotic arrest of primary fibroblast followed either by cell death or checkpoint slippage and aneuploidy. The latter will hamper the potential use of Plk1 inhibitors for the depletion of proliferating cells from differentiated cell cultures for cell therapy and might be a concern in general. Nevertheless, as shown here, it could be useful in cell cultures for *in vitro* applications.

## Supporting Information

File S1 Cardiomyocytes were treated with BI2536 and then recorded by video imaging. Note the presence of rounded mitotic fibroblast, which are absent in the control culture (File S2).
(9.82 MB MOV)

File S2 Video imaging of a control culture of cardiomyocytes (without treatment).
(8.53 MB MOV)
